# A multiarticulate pediatric prosthetic hand for clinical and research applications

**DOI:** 10.3389/frobt.2022.1000159

**Published:** 2022-10-28

**Authors:** Marcus A. Battraw, Peyton R. Young, Wilsaan M. Joiner, Jonathon S. Schofield

**Affiliations:** ^1^ Department of Mechanical and Aerospace Engineering, University of California, Davis, Davis, CA, United States; ^2^ Departments of Neurobiology, Physiology and Behavior, University of California, Davis, Davis, CA, United States; ^3^ Department of Neurology, University of California, Davis, Davis, CA, United States

**Keywords:** pediatric prostheses, research platform, upper limb, multiarticulate prosthesis, grasping

## Abstract

Although beginning to emerge, multiarticulate upper limb prostheses for children remain sparse despite the continued advancement of mechatronic technologies that have benefited adults with upper limb amputations. Upper limb prosthesis research is primarily focused on adults, even though rates of pediatric prosthetic abandonment far surpass those seen in adults. The implicit goal of a prosthesis is to provide effective functionality while promoting healthy social interaction. Yet most current pediatric devices offer a single degree of freedom open/close grasping function, a stark departure from the multiple grasp configurations provided in advanced adult devices. Although comparable child-sized devices are on the clinical horizon, understanding how to effectively translate these technologies to the pediatric population is vital. This includes exploring grasping movements that may provide the most functional benefits and techniques to control the newly available dexterity. Currently, no dexterous pediatric research platforms exist that offer open access to hardware and programming to facilitate the investigation and provision of multi-grasp function. Our objective was to deliver a child-sized multi-grasp prosthesis that may serve as a robust research platform. In anticipation of an open-source release, we performed a comprehensive set of benchtop and functional tests with common household objects to quantify the performance of our device. This work discusses and evaluates our pediatric-sized multiarticulate prosthetic hand that provides 6 degrees of actuation, weighs 177 g and was designed specifically for ease of implementation in a research or clinical-research setting. Through the benchtop and validated functional tests, the pediatric hand produced grasping forces ranging from 0.424–7.216 N and was found to be comparable to the functional capabilities of similar adult devices. As mechatronic technologies advance and multiarticulate prostheses continue to evolve, translating many of these emerging technologies may help provide children with more useful and functional prosthesis options. Effective translation will inevitably require a solid scientific foundation to inform how best to prescribe advanced prosthetic devices and control systems for children. This work begins addressing these current gaps by providing a much-needed research platform with supporting data to facilitate its use in laboratory and clinical research settings.

## 1 Introduction

It is estimated that congenital upper limb differences occur in up to 1 in 500 live births (Giele et al., 2001), and those with unilateral congenital below-elbow deficiencies typically present malformations amenable to prosthesis prescription. These children will have one typical upper limb and one that ends below the elbow, at the level of the proximal or mid-forearm (Edmonds et al., 1981; Krebs and Fishman, 1984; Davids et al., 2006). Prosthesis prescription for these children is a complex challenge, and presently 35%–45% of prescribed upper limb pediatric prostheses will be abandoned (Biddiss and Chau, 2007). Regardless of age, factors that affect prosthesis adoption are related to the device offering sufficient function while promoting healthy social interactions (Vasluian et al., 2013). The high rate of pediatric prosthesis abandonment suggests that current devices fall short of achieving these demands and specific reasons for abandonment include the lack of useful function offered by the device (Postema et al., 1999; Wagner et al., 2007; Vasluian et al., 2013), device weight (Egermann et al., 2009; Vasluian et al., 2013), discomfort (Postema et al., 1999; Wagner et al., 2007), and social aspects related to device cosmesis (Postema et al., 1999; Vasluian et al., 2013; Franzblau et al., 2015; Oliver et al., 2018).

Standard of care pediatric prostheses provide limited functionality, typically offering only a single degree-of-freedom open/close grasping function. This is a stark departure from the immense dexterity of an intact hand that moves with 27 degrees of freedom (Agur and Lee, 1999), and the 6–9 common hand grasp movements (pulp pinch, cylindrical grasp, among others) that have been shown to account for nearly 80% of grasping movements when performing activities of daily living (Zheng et al., 2011; Feix et al., 2016). In recent years, multi-articulating motorized prosthetic hands for adults have become increasingly available. These assistive devices offer adults significant functional benefits by providing a multitude of hand grasp configurations (Belter et al., 2013). Beyond their added function, an additional advantage inherent to their hand-like designs is the anthropomorphic or more life-like appearances when compared to their hook or grasper-style counterparts. Similarly, dexterous devices have begun to emerge for children, namely, the Vincent Young three (Vincent Systems, Karlsruhe, Germany) which is sized for an 8-year-old and offers up to 13 individual grasp configurations, or the Hero Arm (Open Bionics, Bristol, United Kingdom) which offers children 8 years and older six grasp configurations.

As dexterous pediatric prostheses continue to emerge there remain many unanswered questions such as which control techniques may be most effective in operating these devices, the degree to which children can use the newly available dexterity for improved functional outcomes, and how best to translate many effective innovations for adults to meet the unique demands of children (Battraw et al., 2022a). For example, it is not known which grasping motions may be most effective to support age-appropriate daily activities and childhood play. Additionally, it is unknown how conventional adult muscle-based prosthesis control (surface EMG) may be translated to this population given that many were born with their limb difference and their affected muscles have never actuated an intact limb (Battraw et al., 2022b). Although control of dexterous prostheses for adults with congenital upper limb deficiencies has been investigated (Kryger et al., 2011), it is uncertain how these findings may translate to developing children. Furthermore, limited work has been done to illustrate changes in cortical activation during prosthesis control (Da Paz and Braga, 2007; Copeland et al., 2021). Addressing these knowledge gaps requires rigorous scientific investigations and supporting research platforms; hardware such as dexterous child-sized prostheses with open access to its programming and the mechanical capabilities to interact with daily objects to perform clinical or research-based activities. While there are no robust pediatric research platforms, there are numerous experimental or non-clinical pediatric prostheses that have been reported in literature; however, data characterizing their use, functional capabilities, and effectiveness remain sparse (Ten Kate et al., 2017). Furthermore, researchers and clinicians often have limited access to these devices as they are not commercially available, and few are released open-source such that they can be fabricated and programmed by individuals outside of their development teams.

Our objective was to develop a child-sized multi-grasp prosthesis that may serve as a robust research platform to address many of the critical gaps in translating dexterous upper limb prostheses to pediatric populations. In anticipation of an open-source release, we performed a comprehensive set of benchtop and validated functional tests manipulating common objects to quantify the performance of our device. Here we present the development of a cable-driven, underactuated, adaptive grasp, multi-articulate pediatric hand termed the Bionic Engineering and Assistive Robotics Pediatric Assistive Ware (BEAR PAW). The mechanical and electrical characteristics of individual digit articulation and seven commonly used hand grasps (Feix et al., 2016) are presented, followed by the functional performance benchmarked against other multi-grasp devices using an established assessment protocol (Llop-Harillo et al., 2019).

## 2 Materials and methods

We performed three tasks that were designed to develop, characterize, and evaluate the performance of the BEAR PAW. Design criteria were derived to inform the development and fabrication of our pediatric device. We performed benchtop testing to evaluate the device’s mechanical and electrical characteristics, and we evaluated the BEAR PAW while grasping common objects to benchmark its performance against other comparable adult devices.

### 2.1 Pediatric prosthetic hand criteria

In developing a robust research platform, delivering a device capable of achieving multiple hand grasp configurations to a similar degree of dexterity as current research-based adult devices was the crux of the challenge. The size of the device was an important first step to consider, as this directly impacted the feasibility of device development. As emerging dexterous devices have been targeted to no younger than the 8-year-old population and off the shelf componentry is limited in size, the minimum age of eight provides us with an ideal size constraint. Furthermore, to achieve comparable dexterity, individual digit actuation was needed along with an active opposable thumb. Weight was another important consideration during device development because children do not yet have the strength of an adult (Egermann et al., 2009). Even in a research setting, it is important to carefully consider this constraint as fatigue, soreness, and/or discomfort can significantly diminish a child’s engagement with experimental activities. Here, the mass of an Ottobock Electrohand 2000 for children 8–13 years old was used as a baseline for comparison (130 g) as it is among the lightest commercially available terminal devices for children. Additionally, the force output of the device was of high importance as in biological hands, it has been shown that most hand grasping configurations on average hold objects less than 500 g in weight during most activities of daily living (Feix et al., 2016) making this an ideal design target value for a pediatric prosthesis. Further, the time to fully close the hand was set to be less than 1 s , reflecting values found among commercially available prosthetic systems (Vujaklija et al., 2016). Finally, a budget value of less than $1000 for parts was selected to promote the accessibility of our system to other research laboratories. A detailed summary of the design criteria is outlined in Table 1.

**TABLE 1:** Pediatric research platform design criteria.

Design requirement	Specification metric	Quantitative value
Size	Anatomical proportions	8-year-old child
Mass	Low mass	<130 g
Inexpensive	Low cost	< $1000
Degrees of actuation	Digit actuation and thumb opposition	6 degrees of actuation
Active actuation	Servo control	Servo motors
Electronics	Compact design	Enclosed in hand
Extended operation	Continuous power	Grid power
Control	Ease of actuation	Bluetooth protocol
Ease of use	High usability	Graphical interface
Finger speed	Time to close	<1 s
Load	Target mass	500 g

### 2.2 Mechanical and electrical performance

#### 2.2.1 Experimental setup

We characterized the mechanical and electrical performance of the BEAR PAW while performing a set of the most frequently used generalized hand grasps along with individual digit actuations. Feix et al. (2016) suggests that the vast majority of human object manipulations are accomplished using 33 different grasp types which can be simplified to 17 generalized hand grasp configurations. This simplification can be made when considering that objects of different shapes and sizes may actually require the hand to move in similar ways, just to differing degrees of hand closure (Feix et al., 2016). This is a relevant consideration as the BEAR PAW is programmed to conform to objects regardless of their size. Of the 17 generalized hand grasps some are used far more frequently than others, and a subset of seven accounts for 80% of total activity (Table 2). Furthermore, these seven grasps also accounted for over 80% of the time duration in which a hand is used to grasp objects in daily living. Table 2 shows the top seven generalized hand grasps that were used to characterize the BEAR PAW’s performance.

**TABLE 2:** Top seven generalized hand grasp configurations, percent frequency (Freq), and duration (Dur).

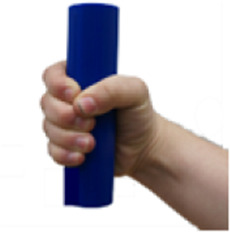	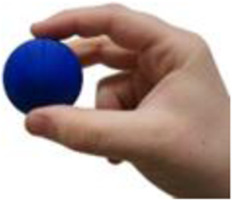	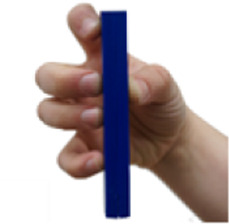	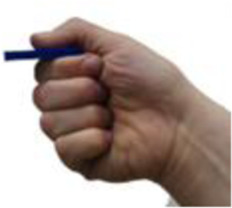	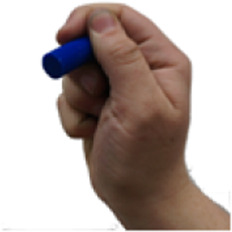	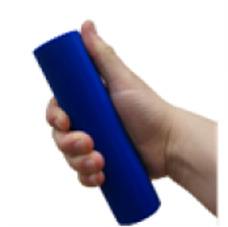	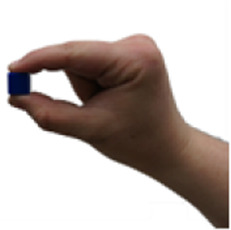
1. Cylindrical Grip	2. Tripod Pinch	3. Prismatic 4 Finger	4. Lateral Pinch	5. Lateral Tripod	6. Hook Grip	7. Pulp Pinch
Freq: 21.6%	Freq: 14.8%	Freq: 11.3%	Freq: 10.5%	Freq: 10.4%	Freq: 6.8%	Freq: 4.8%
Dur: 30.5%	Dur: 10.4%	Dur: 26.9%	Dur: 6.9%	Dur: 5.1%	Dur: 5.1%	Dur: 2.7%

A set of six custom manipulanda were designed and fabricated to measure the force characteristics of the BEAR PAW while performing the seven grasp configurations and individual digit actuations. These consisted of a series of 3D printed enclosures that housed one to two calibrated 8 mm diameter SingleTact capacitive force sensor(s) with a range of 10 N (SingleTact CS8-10, PPS United Kingdom Limited, Glasgow, United Kingdom) (Table 3).

**TABLE 3:** The different manipulanda used to characterize the force output of the BEAR PAW for individual finger articulation and common generalized hand grasp configurations. The hand grasp (HG) used and the number of sensors (NS) for each manipulandum are noted and each square on the blue background is 1 cm by 1 cm.

Top view	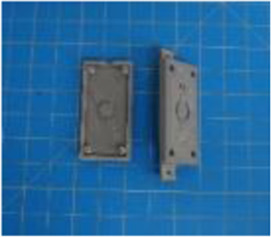	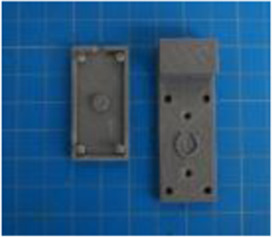	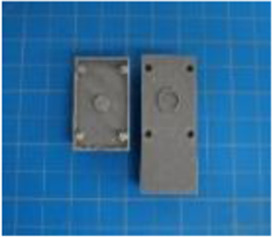	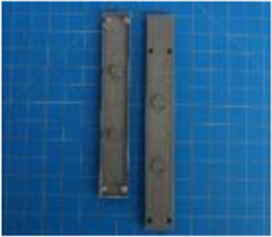	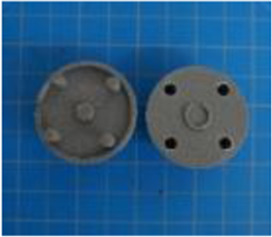	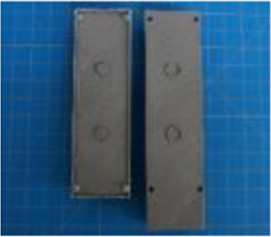
Isometric	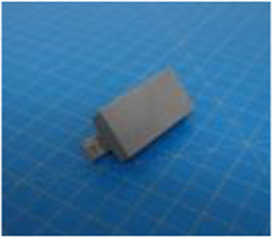	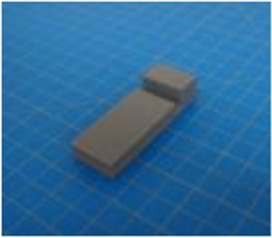	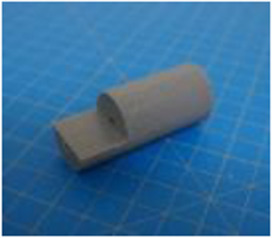	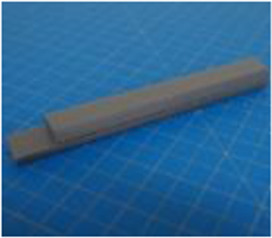	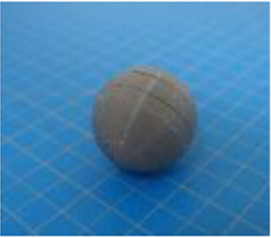	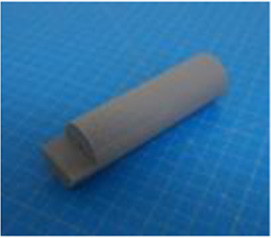
Description	HG: Finger Articulation	HG: Pulp Pinch, Lateral Pinch	HG: Lateral Tripod	HG: Prismatic 4 Finger	HG: Tripod Pinch	HG: Hook Grip, Cylindrical Grip
	NS: 1	NS: 1	NS: 1	NS: 2	NS: 1	NS: 2

**FIGURE 1:**
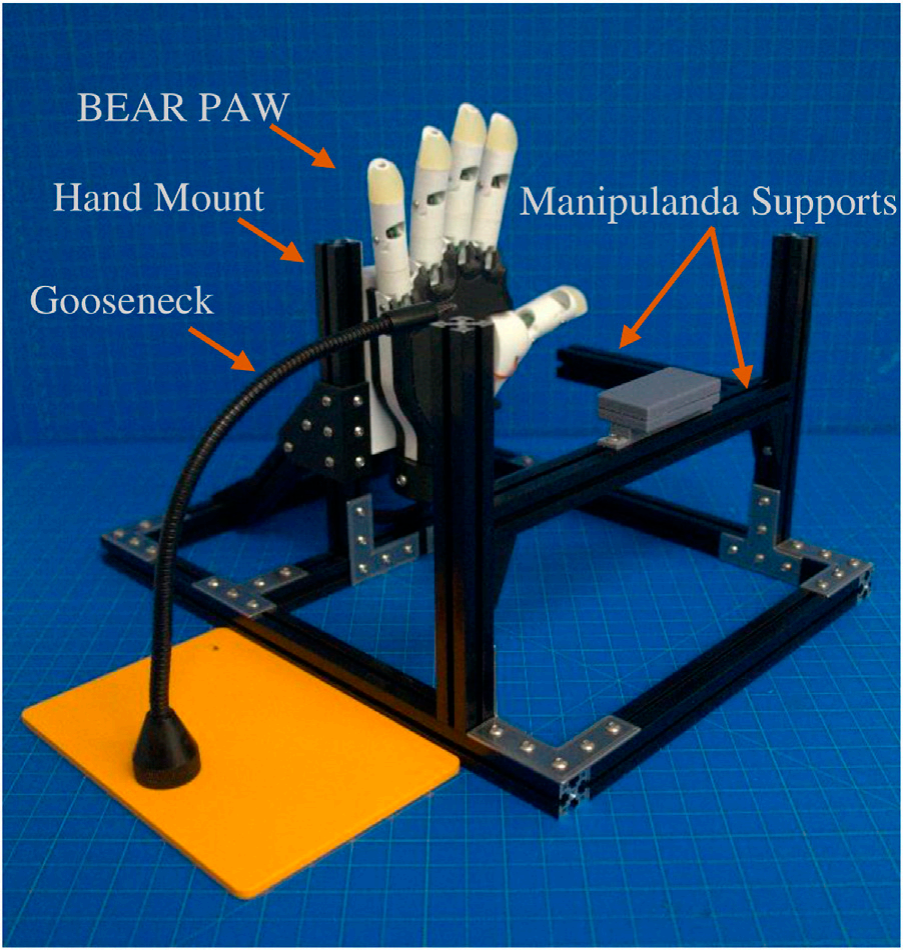
Depicts the testing platform for the BEAR PAW. It illustrates the hand mount used to hold the BEAR PAW stable during testing, the gooseneck which strategically held manipulanda, and the MakerBeam platform which supported the manipulandum used for individual digit articulation.

A testing platform was assembled with 15 × 15 mm MakerBeam and included a custom 3D printed mount for the BEAR PAW. The platform was designed to fixate the BEAR PAW which allowed for repeated consistent testing of the various hand motions during data collection. Additionally, the platform accommodated the set of manipulanda to capture the mechanical force output. These were either mounted to the platform or on an external gooseneck for strategic object placement (Figure 1).

Beyond the mechanical force measurements obtained using the manipulanda, the electrical characteristics of the BEAR PAW were recorded during testing. This included capturing the current obtained with an ACS723 current sensor which recorded the current load of the BEAR PAW’s servo motors during the experimental procedure. Further, the voltage across the servo motors during actuation was recorded. Lastly, to synchronize the data during post-hoc analysis a timing voltage was used. An Arduino script was written to actuate the BEAR PAW and the voltage values produced from the force, servo current, servo voltage, and time voltage were passed into a National Instruments USB-6210 data acquisition system sampling at 4000 Hz . This data was stored for further analysis in a table format using a MATLAB (The MathWorks, Inc., Natick, MA) script.

#### 2.2.2 Experimental procedures

**FIGURE 2:**
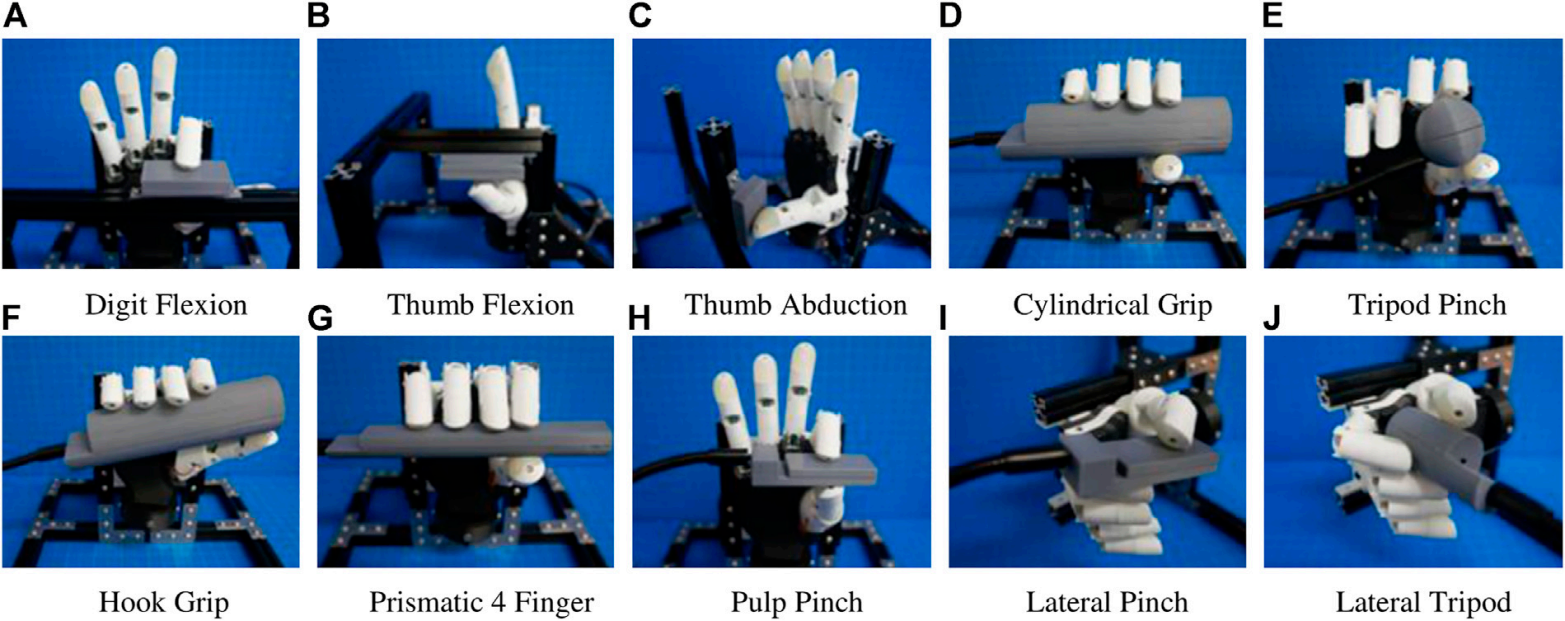
Depicts the BEAR PAW during grasp actuation on the various manipulanda. **(A)** Represents individual digit articulation for digits 2–5 and **(B–C)** represents both thumb palmar abduction and flexion. **(D–J)** Shows each manipulandum used for the seven common generalized hand grasp configurations.

The BEAR PAW was tested to determine the mechanical and electrical performance when completing individual digit and grasp actuations. In both configurations, the BEAR PAW was mounted to the testing platform to assess the grasping movements shown in Figure 2. To test individual digit flexions, the manipulandum was placed at a fixed distance and was then aligned with the digit so that it would press down on its center. For each hand grasp configuration, the appropriate manipulandum was attached to the gooseneck (Figure 1) and was strategically placed in front of the BEAR PAW (Figure 2).

Testing was performed in accordance with ANSI/ISA testing protocols (ANSI/ISA Process Instrumentation Terminology, 1979). The test procedure consisted of performing single-digit actuations and the hand grasp configurations 10 times each. Here one cycle consisted of the BEAR PAW actuating for a total of 5 s to grasp/load the manipulandum and then unload it. The current from the servo motors, voltage across the servo motors, force applied to the manipulandum, and a reference voltage used for data synchronization, were measured and stored for each testing cycle. Together, these data allowed for post-hoc calculations relating force, current, and power each time the BEAR PAW performed a grasping movement (see below).

#### 2.2.3 Data analysis

A separate MATLAB script was written to read the stored data for analysis. First, the voltage output from the force sensor(s) in the manipulanda was converted to force using the line of best fit for each of the calibrated sensors (User Manual: SingleTact Miniature Force Sensors. Rev 2.3, 2017). Further, in the case of two force sensors, a point load was assumed at each sensor, and data were summed together after conversion to include the total force value. The voltage from the current sensor output was converted to amperes using the provided IC sensitivity of 400 mV/A (High Accuracy, Galvanically Isolated Current Sensor IC With Small Footprint SOIC8 Package. Rev 4, 2018). Finally, Watt’s law was used to calculate power draw from the measured voltage across the servo motors and the corresponding current.

To align data across the 10 trials a reference timing voltage was used, during the 5 s of actuation, that was set to low until the BEAR PAW began to actuate at which point it was set to high. Once this occurred, 1 s of the data directly after the high was omitted followed by 2.5 s of recorded data to ensure that the BEAR PAW was fully actuated on the manipulanda. For individual digit actuations and generalized hand grasp configurations these 2.5 s were averaged for a total of 10 values, one per each actuation cycle. Here, the mean and standard deviation of these measures were obtained. Measures obtained during the flexion of digits 2–5 were averaged together as these fingers are identical in size and mechanical design. Values for thumb flexion and opposition were captured separately. Additionally, all generalized hand grasp configuration measures were averaged on an individual grasp basis.

### 2.3 Hand assessment protocol

#### 2.3.1 Experimental setup

To assess the BEAR PAW’s functional capabilities, we used the validated Anthropomorphic Hand Assessment Protocol (AHAP) (Llop-Harillo et al., 2019). The protocol consists of eight different grasp types of which there are three different objects associated with each. The eight grasps are Hook Grip, Spherical Grip, Tripod Pinch, Extension Grip, Cylindrical Grip, Diagonal Volar Grip, Lateral Pinch, and Pulp Pinch. Furthermore, there are two postures—Index Pointing and Platform—for a total of 26 objects that must be grasped and/or maintained. A further explanation of the objects used during the AHAP test can be found in (Llop-Harillo et al., 2019) and a subset of these objects are depicted in the results section.

**FIGURE 3:**
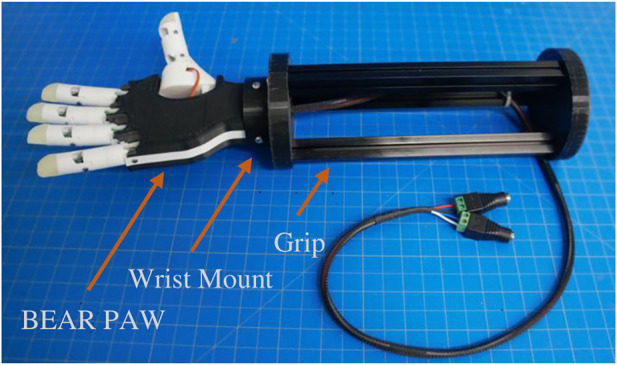
Depicts the testing rig used to perform the Anthropomorphic Hand Assessment Protocol, highlighting the BEAR PAW, wrist adapter mount, and forearm frame grip.

We preprogrammed grasp configurations into the BEAR PAW in accordance with the definitions used in (Llop-Harillo et al., 2019). These definitions explained the proper posture for each grasp and indicated the correct contact between an object and various locations on a robotic hand. With these definitions, the BEAR PAW’s hand grasp configurations were created in software by adjusting individual digit positions which allowed for it to appropriately conform to the test objects. This was achieved using a custom developed graphical user interface (GUI) that allowed the investigators to fine-tune the digit movements for each grasp configuration using virtual buttons and knobs. The final settings were stored, and the GUI offered the ability to then simply press a virtual button to actuate the final grasping configurations. To perform the AHAP protocol a testing rig was developed which consisted of the BEAR PAW mounted to a forearm frame through a wrist mount (Figure 3) which could then be held by the investigator to perform necessary object manipulations.

#### 2.3.2 Experimental procedures

The AHAP test required that 26 test objects be manipulated 3 times which was then repeated by three test investigators (Llop-Harillo et al., 2019). Replicating the test with 3 separate investigators is the standard AHAP procedure and ensures that collected data accounts for the minor potential variability in the way objects may be manipulated. Here, investigators included laboratory personnel who acted as the lead investigator and test investigators. Prior to conducting the protocol the test investigators were instructed by the lead investigator as to the correct hand grasp for the object and were allowed to familiarize themselves for approximately 1 min (Llop-Harillo et al., 2019). Each trial of the AHAP protocol began with the lead investigator holding 1 of the 26 objects in front of a test investigator in a predefined orientation. The test investigator utilized the GUI to actuate the BEAR PAW to achieve a desired grasp configuration and grasp the object. Afterwards, the lead investigator would release the object such that it was held exclusively by the BEAR PAW. For each grasp type (excluding postures), the BEAR PAW started in the palm faced up direction in which it attempted to hold the corresponding object for 3 s (known as the grasping phase) and then was rotated 180 ° with the palm faced down again attempting to hold the object for 3 s (known as the maintaining phase). The index posture consisted of starting a timer for the grasping phase and stopping it after 3 s for the maintaining phase. Additionally, the platform posture only involved the grasping phase which entailed holding a plate for 3 s. The grasping and maintaining phases for each grasp type and posture are further described in (Llop-Harillo et al., 2019).

#### 2.3.3 Data analysis

During the grasping and maintaining phases for each object, the lead investigator scored the BEAR PAW’s performance (Llop-Harillo et al., 2019). Accordingly, a score of one was received if the object was held with the specified grasp for the allotted time. A score of 0.5 was received if the BEAR PAW held the object for the designated time but was done with a different grasp and 0 was received if it could not hold the object. Then, while the BEAR PAW performed the maintaining portion, if there was no movement of the object with respect to the hand over the time constraint a score of one was awarded. If the object moved but did not drop then a score of 0.5 was received and a score of 0 was received if it was not able to maintain the object. The BEAR PAW’s raw AHAP scores are provided in the supplementary material.

These scores were then used to compare the BEAR PAW’s grasping and maintaining abilities to previously published values from four research-focused adult prosthetic hands performing the same experimental procedure (Llop-Harillo et al., 2020). These four adult hands (Dextrus, IMMA, InMoov, and Limbitless) were all underactuated systems with a range from 14 to 17 degrees of freedom and 1–6 degrees of actuation (Llop-Harillo et al., 2020). Here, scores obtained from the BEAR PAW and the four adult prosthetic hands were separated based on which phase (grasping or maintaining) the prosthetic hand was in. The scores for each prosthetic hand were further separated into 10 categories for grasping and nine categories for maintaining in accordance with the grasp type/posture. These scores were aggregated across the three test investigators such that individual grasping and maintaining comparisons could be made between the BEAR PAW and the four adult prosthetic hands.

To accommodate the ordinal (non-parametric) AHAP scoring data, statistical analyses were conducted using a Mann-Whitney U test to perform pairwise comparisons between the BEAR PAW and each of the four adult prosthetic hands (for the 10 grasps and nine postures, 40 and 36 comparisons, respectively). For each comparison, the null hypothesis H_o_ was that the central tendency or median score of both the BEAR PAW and the adult hand that was being compared are not significantly different for a given grasp. A confidence interval of 95% was selected and *p* < 0.05 was taken to indicate statistical differences.

## 3 Results

### 3.1 Pediatric prosthetic hand

**FIGURE 4:**
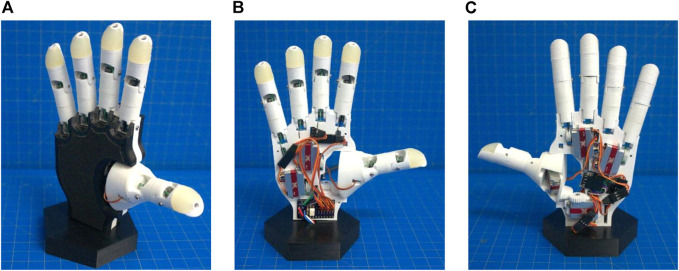
The BEAR PAW: A pediatric multiarticulate prosthetic hand with six degrees of actuation and programmable hand grasp configurations. Shown in an isometric **(A)**, front **(B)**, and back **(C)** view.

The BEAR PAW is a multi-articulating pediatric prosthetic hand developed in the computer automated design software SolidWorks 2020 and fabricated with a SigmaX R19 3D Printer using PLA material. The BEAR PAW utilizes a 3.3 V Arduino Pro Mini with an ATmega328 microcontroller, HC-05 wireless Bluetooth module, and a custom breakout board to interface with the six KST-X08 series servo motors. Further, it internally houses its electronics, has six independently programmable degrees of actuation, is an under-actuated system with 11 degrees of freedom, and is therefore capable of a multitude of common grasping movements. In summary, the BEAR PAW is a dexterous pediatric prosthetic hand that was designed using off-the-shelf components, highly accessible design and fabrication techniques, and open access to programming which includes a graphical user interface for intuitive control. A detailed depiction of the BEAR PAW is presented in Figure 4 and a detailed list of its performance characteristics is supplied in Table 4.

**TABLE 4:** BEAR PAW achieved specifications. *Values obtained as explained in the Materials and Methods subsection on Mechanical and Electrical Performance and the detailed analysis are provided in the corresponding Results section. †The STL files and assembly guide can be found at https://github.com/BEAR-Labs/BEAR-PAW.

Specification	Achieved value
Size/Appearance	
Anatomical proportions	8 years old child
Electrical	
Operating voltage	5 V
Actuation power	3.388–8.718 W*
Mechanical	
Time to grasp	0.67 s
Force	0.424–7.216 N*
Number of actuators	6
Type of actuators	Servo motors
Actuation type	Underactuated
Actuation mechanism	Tendon driven
Range of motion	
Degrees of freedom	11
Digit 2–5 flexion	120 degrees
Thumb flexion	90 degrees
Thumb abduction	90 degrees
Control	
Able-bodied control	Graphical interface
Communication	Bluetooth, UART
Weight	
Mass	177 g
Ease of access	
Cost	500 USD
Componentry	Off the shelf
STL Files	Available online†
Assembly guide	Available online†

**FIGURE 5:**
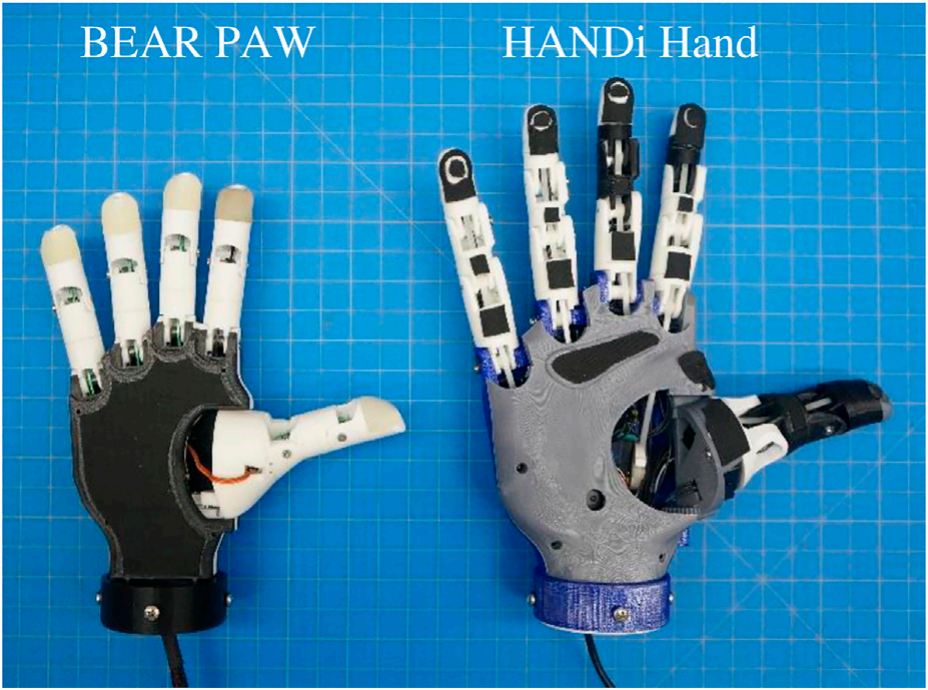
A size comparison between the BEAR PAW, a pediatric prosthetic hand, and the HANDi Hand, an adult prosthetic hand. Left shows the BEAR PAW and right shows the HANDi Hand. Each square in the background is 1 cm by 1 cm.

The BEAR PAW’s design and development was inspired by the HANDi Hand and was sized to 50th percentile 8-year-old male and female anthropometric hand data (Figure 5) (Snyder et al., 1977; Brenneis et al., 2017; Cheng et al., 2019). Similar to the HANDi Hand the BEAR PAW is accessible to researchers and clinicians, and provides open source 3D printable files, a bill of materials, assembly instructions, microcontroller code, and GUI which can be found *via* https://github.com/BEAR-Labs/BEAR-PAW.

### 3.2 Mechanical and electrical performance

**FIGURE 6:**
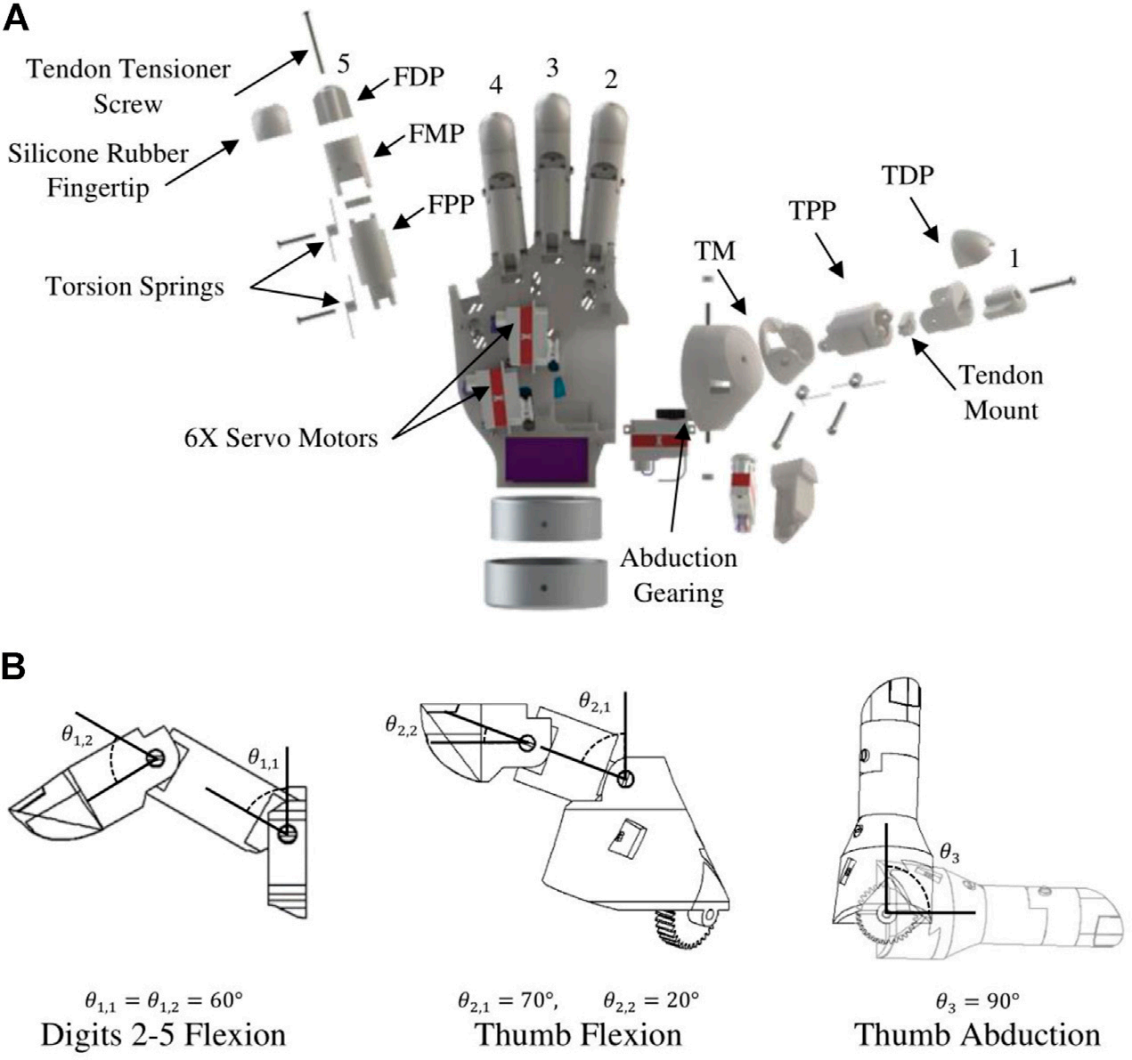
A detailed illustration of the mechanical features of the BEAR PAW. **(A)** Shows an exploded view of individual digits 1–5 highlighting key components of the mechanical design. **(B)** Provides the range of motion for each degree of actuation. Digits 1–5 are labeled with acronyms: finger distal phalanx (FDP), middle phalanx (FMP), proximal phalanx (FPP), thumb distal phalanx (TDP), proximal phalanx (TPP), and metacarpal (TM).

The BEAR PAW uses an underactuated tendon-driven design in each digit to achieve flexion, and torsion springs incorporated into each joint to return digits to their extended position when not being actuated (Figure 6A). Here flexion is caused by a servo motor rotating a pulley to which a tendon is attached. One challenge with conventional tendon-driven actuation is managing the slack that may present in the tendon. Therefore, we developed a tensioning mechanism in which a tensioner screw translates a tendon mount to compensate for the slack. Moreover, the digits 1–5 are all actuated and controlled independently; while thumb abduction uses gearing for motion and is also actuated independently.

The anatomical design of digits 2–5 for the BEAR PAW included the distal, middle, and proximal phalanx where the distal and middle are coupled to accommodate the small size required of a pediatric hand. The range of motion for these digits during flexion (while not contacting objects) was approximately 60° for the proximal and middle-distal phalanx. Further, digit 1 included the distal and proximal phalanx along with the thumb metacarpal. During thumb flexion a 70° range of motion for the proximal and 20° for the distal was achieved, respectively. Finally, thumb abduction had a 90° range of motion (Figure 6B).

The measured force outputs for the BEAR PAW while performing the seven grasping configurations and individual digit articulations ranged from 0.424 N to 7.216 N. The maximum value of 7.216 N was achieved during Cylindrical Grip while the minimum value of 0.424 N was achieved during the Lateral Pinch (Table 5). The electrical performance ranged from 0.675 to 1.789 A and 3.388–8.718 W across the different grasp configurations. The minimum values of 0.675 A and 3.388 W corresponded to the individual digit flexion of digits 2–5. The maximum values of 1.789 A and 8.718 W were achieved from the Cylindrical Grip which also achieved the highest grasping forces (Table 5).

**TABLE 5:** BEAR PAW’s mechanical and electrical characteristics for the six degrees of actuation and the top seven generalized hand grasp configurations. *Hook Grip and Diagonal Volar Grip have the same gross hand motion, yet in the AHAP test these are considered separate motions which include a different set of objects.

Motion posture	Motion picture	Mechanical and electrical characteristics		
		*Force (Newtons)*	*Current (Amperes)*	*Power (Watts)*
Digits 2–5 Flexion	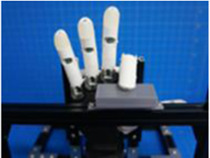	1.709 ± 0.076	0.675 ± 0.069	3.388 ± 0.343
Thumb Flexion	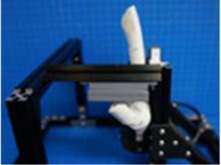	0.761 ± 0.042	0.751 ± 0.002	3.763 ± 0.010
Thumb Abduction	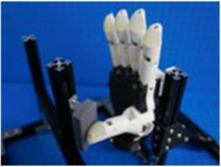	2.454 ± 0.069	0.729 ± 0.003	3.656 ± 0.014
Cylindrical Grip	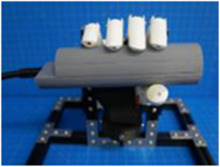	7.216 ± 0.578	1.789 ± 0.052	8.718 ± 0.242
Tripod Pinch	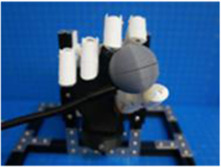	2.989 ± 0.253	1.433 ± 0.035	7.030 ± 0.166
Prismatic 4 Finger	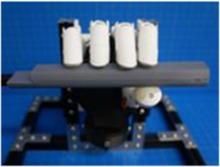	5.714 ± 0.190	1.644 ± 0.068	8.011 ± 0.316
Lateral Pinch	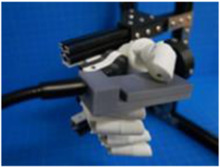	0.424 ± 0.011	0.841 ± 0.008	4.115 ± 0.042
Lateral Tripod	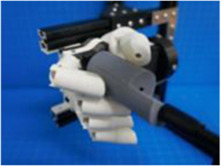	0.629 ± 0.072	0.840 ± 0.005	4.097 ± 0.024
Hook Grip/Diagonal Volar Grip*	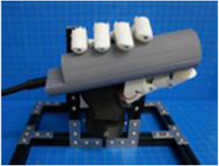	1.415 ± 0.158	1.083 ± 0.020	5.276 ± 0.109
Pulp Pinch	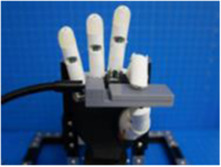	2.043 ± 0.025	0.949 ± 0.004	4.649 ± 0.020

### 3.3 Hand assessment

**FIGURE 7:**
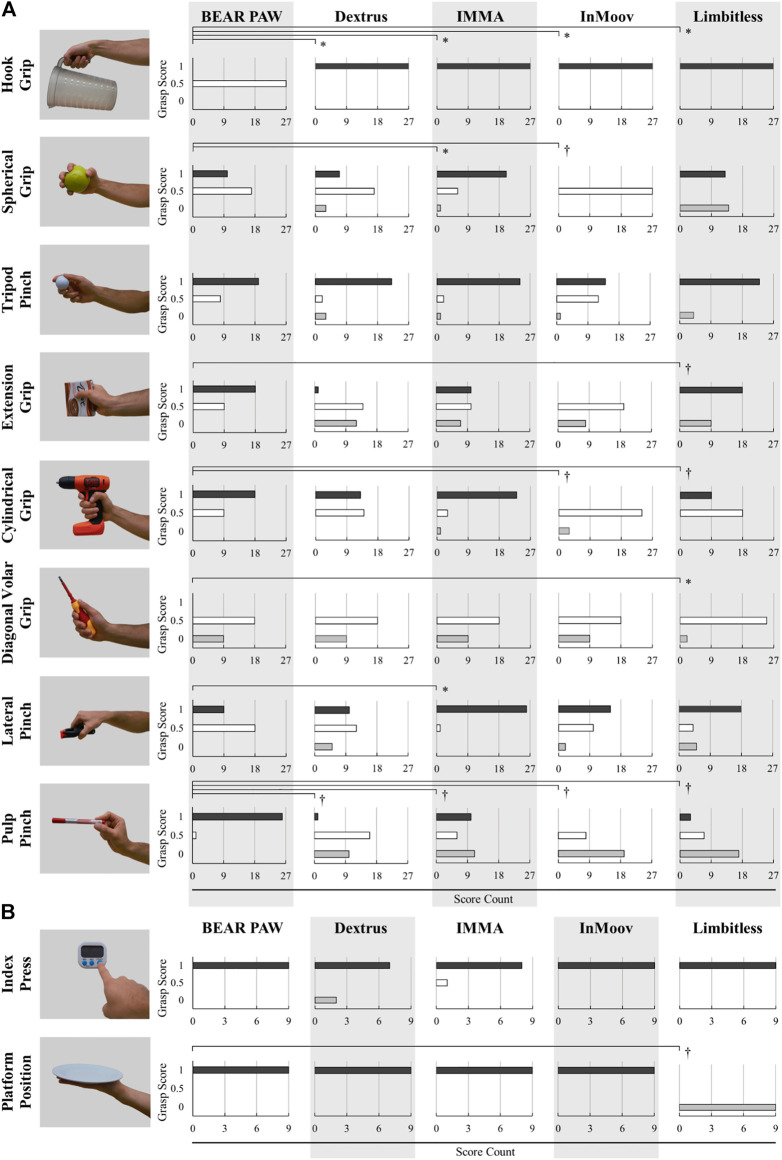
BEAR PAW grasping comparison scores for the 10 different **(A)** grasp types and **(B)** postures across the four adult hands. For each grasp type/posture, the number of times each hand scored a 1, 0.5, or 0 was plotted. *Represents when the BEAR PAW performed statistically worse. †Represents when the BEAR PAW performed statistically better.

When statistically comparing the BEAR PAW’s grasping performance to published values of the four research-focused adult prosthetic hands, its performance scored better or equivalent for 33 of the 40 comparisons made (10 grasps for four adult hands) (Figure 7). Further, 31 times out of 36 the BEAR PAW performed statistically better or equivalent during the maintaining phase for the nine grasp type/posture categories (Supplementary Material). That is, minor differences exist between the BEAR PAW and the four adult prosthetic hands when comparing grasping and maintaining capabilities.

For the grasping phase of the AHAP test, the statistical analysis showed the BEAR PAW performed significantly better a total of 9 times across the four adult prosthetic hands. Further, 24 times there were no statistically significant differences observed during the grasping phase. Finally, when comparing the BEAR PAW to each adult prosthetic hand the analysis showed statistically worse performance for seven of the grasp types/postures. A detailed analysis of the grasping comparisons from the BEAR PAW to each of the four adult prosthetic hands across the 10 grasp types/postures can be seen in Figure 7. In this figure, the number of times the hand scored a 1, 0.5, or 0 for a grasp type/posture was tallied and plotted. Further, this figure depicts a subset of the 27 objects used in the AHAP test as a reference.

The statistical analysis for the maintaining phase of the AHAP test showed significant differences between the BEAR PAW and each of the four adult prosthetic hands. There was a significantly better performance for 16 grasp types/postures, 15 were shown to have no significant differences, while five showed statistically worse performance. The detailed statistical comparison for the maintaining phase of the test can be viewed in the Supplementary Material.

For both the grasping and maintaining phases of the AHAP test, the BEAR PAW performed significantly worse for the Hook Grip a majority of the time with only one comparison that showed no significant difference. Additionally, the BEAR PAW performed significantly better for the Pulp Pinch across all adult prosthetic hands. Finally, for the maintaining phase, the Cylindrical Grip of the BEAR PAW showed significantly better results than the other prosthetic hands. In summary, the BEAR PAW performed similarly to the four adult prosthetic hands and in some cases better, making it an effective platform to examine prosthetic control in pediatric populations.

## 4 Discussion

This work presents the design and characterization of a multiarticulate pediatric-sized prosthetic hand that may serve as a robust and accessible research platform. The series of benchtop tests performed in this study provide a benchmark analysis of the device. Its performance, when compared to research-focused adult prosthetic hands, suggests that the BEAR PAW has the potential to serve as a useful tool in exploring the multitude of questions and unique challenges surrounding the effective translation of advanced mechatronic prostheses to children.

Multiple, clinically relevant design criteria were employed to inform the design and fabrication of the BEAR PAW and to ensure its utility as a research platform. These criteria included a size and weight limit, device dexterity, control methods, and accessibility. Intuitively, these criteria are interconnected and directly influence one another. A prominent example of this relationship is as dexterity increases, the number of actuators must also increase, and with that, the weight and the compact size of the device become difficult to address. This issue is vital to the BEAR PAW as it is a highly dexterous device, that is, tailored to conform to the anthropomorphic nature of an 8-year-old child to meet the need for a child-sized dexterous device. Although it is possible to develop smaller dexterous devices targeted at a younger population (less than 8 years old), commercial devices have yet to emerge, and it is unlikely a research platform with off the shelf componentry could exist as the next step to miniaturization would require hardware development. Furthermore, while the BEAR PAW exceeded the target weight limit of 130 g (weighing 177 g), the device weighs less than comparable dexterous pediatric hands such as the Hero Arm hand [280–345 g, (Hanger Clinic, 2019)], and is designed to be used in a research setting, allowing the researcher opportunities to make necessary adjustments to test procedures thereby minimizing subject fatigue.

As children’s motor systems are still developing and they are often still exploring interactions within their environments, a more dexterous device is vital to allow them to interact with objects in different ways using a multitude of hand gestures (Battraw et al., 2022b). The BEAR PAW can achieve similar dexterity to that of the comparable adult prosthetic hands, providing researchers control over individual digit movements and thus, the ability to explore the effects of providing users multiple grasping configurations. Further, the BEAR PAW can accommodate multiple communication protocols and incorporates affordable off-the-shelf componentry to provide ease of use and accessibility to research groups. The 3D printable files, assembly instructions, bill of materials, and necessary code are openly available to further facilitate this access (https://github.com/BEAR-Labs/BEAR-PAW). Well-documented and tested open-source pediatric hands are scarce making experimentation with these devices difficult. Furthermore, current commercially available devices inhibit researchers’ ability to manipulate device hardware/software to push the boundaries of the current state of pediatric prostheses. Here, we begin to address this gap by disseminating an open-source research platform with documented performance characteristics and benchmarking it to well-known adult research devices.

Feix et al. (2016) suggest that the majority of objects that adults commonly manipulate in daily life do not exceed 500 g, and the grasping force of the hand is largely driven by the mass of the object. The BEAR PAW achieved a maximum grasping force output of 7.216 N which exceeded the typical force required to statically grasp a 500 g object (Feix et al., 2016). This maximum force output was obtained from the Cylindrical Grip configuration, which was anticipated, as all the digits actuated around the object to perform the grasp thereby utilizing the combined outputs of all servo motors. Conversely, the minimum force output of 0.424 N was associated with the Lateral Pinch grasp and the low force was likely due to the nature of the index finger’s range of motion which was limited by the servo motor to 120 ° . This limited range of motion caused restricted contact between the thumb and index finger. When taken together, the BEAR PAW was able to perform seven common generalized hand grasp configurations successfully, although the device could not achieve the necessary force required to manipulate 500 g objects for every hand grasp configuration. Further design refinements including incorporating high-performance servo motors may be warranted in future work.

Additionally, the electrical characteristics of current and power were tabulated to provide a baseline of electrical performance. It was found during testing that the lowest current and power draw were 0.675 A and 3.388 W, respectively. These results corresponded to the actuation of digits 2–5, which was anticipated as a single digit was being activated and with minimal frictional forces present when compared to individual thumb flexion or geared thumb opposition. Likewise, the value for the maximum current and power draw was 1.789 A and 8.718 W which were recorded from the Cylindrical Grip. Similar to the maximum force, these values were expected as all the servo motors were under load causing an increase in the current and power. Overall, these values provide the necessary information to allow for future untethered battery-operated control.

The AHAP test allowed for the BEAR PAW’s grasping and maintaining ability to be evaluated when manipulating common household objects and benchmarked against the adult prosthetic hands. The objective of performing the comparisons was to validate the BEAR PAW’s performances and viability as a research platform. Here it was found that the BEAR PAW performed similar to or better than comparable adult devices across the test. While it outperformed the tested adult prosthetic hands for Pulp Pinch during both the grasping and maintaining phases, this was likely attributed to the silicone fingertips that allow for increased friction when performing pinch-type manipulations. During the Cylindrical Grip maintaining phase, the BEAR PAW performed better than the other comparable adult prosthetic hands which is intuitive when viewing the mechanical force output of the Cylindrical Grip as it exhibited the highest force output of 7.216 N. However, the BEAR PAW was challenged in performing some functions. The main limitation was the size constraints required to accommodate the pediatric population. Off the shelf micro servo motors that meet these size demands are often restricted in their range of motion, thereby affecting the BEAR PAW’s ability to adequately grasp and maintain certain objects, i.e., the Hook Grip could not fully wrap around smaller objects in the AHAP test. Both the small nature of the design and the limited range of motion affected the AHAP test as certain objects were too big for the BEAR PAW to reach around and too small for the range of motion.

Our data suggest that it is plausible for the BEAR PAW to be used in research and clinical settings to perform tasks and object interactions that may not be overly mechanically demanding such as box and blocks (Mathiowetz and Weber, 1985; Hebert et al., 2014), Jebsen/Taylor hand function (Jebsen et al., 1969), clothespin relocation (Kyberd et al., 2018), and the SHAP test (Light et al., 2002), among others. However, with the exception of the SHAP test (Light et al., 2002), the remaining standardized tests are not designed to challenge the patient to perform more than one grasp type/posture. Although the SHAP test (Light et al., 2002) allows for multiple grasps it uses everyday objects that may not translate effectively to the pediatric population e.g., small hand compared to object size and lack of participant engagement during testing. Therefore, the BEAR PAW can be used to explore the extent to which children can utilize multi-grasp functionality, but like the need for a robust research platform, standardized functional tests that challenge children to perform age-appropriate multi-grasp tasks are also needed. As multi-grasp pediatric devices continue to emerge a rigorous evidence base is required to facilitate clinical adoption and inform the prosthetic approaches to ensure the best functional outcomes for these children. The BEAR PAW provides an accessible, open-source research platform to begin assessing validated outcome measures, refining prosthetic control systems, and examining the degree to which multi-articulating prostheses may make a difference for the users.

## Data Availability

The original contributions presented in the study are included in the article/Supplementary Material, further inquiries can be directed to the corresponding author.
