# Supplementary material for: A multiarticulate pediatric prosthetic hand for clinical and research applications
Source: Front Robot AI. 2022 Oct 28;9:1000159. doi: 10.3389/frobt.2022.1000159 (PMC9651148; doi:10.3389/frobt.2022.1000159)
Supplement: Supplementary file 1 [file Table1.DOCX]

Supplementary Material

**Supplementary Table 1.** Anthropomorphic Hand Assessment Protocol scores from each of the three test investigator for all grasp types/postures over each trial for grasping and maintaining obtained for the BEAR PAW.

| Test Investigator | Trial | Grasp Type/Posture | Object | Grasping | | | Maintaining | | | |
| --- | --- | --- | --- | --- | --- | --- | --- | --- | --- | --- |
|  |  |  |  | Trial 1 | Trial 2 | Trial 3 | Trial 1 | Trial 2 | | Trial 3 |
| 1 | 1 | Hook | Skillet lid | 0.5 | 0.5 | 0.5 | 0.5 | | 0.5 | 0.5 |
|  | 2 | Spherical grip | Plastic apple | 0.5 | 0.5 | 0.5 | 1 | | 1 | 1 |
|  | 3 | Tripod pinch | Large marker | 1 | 0.5 | 0.5 | 1 | | 0.5 | 1 |
|  | 4 | Extension grip | Plate | 1 | 1 | 1 | 0 | | 0 | 0 |
|  | 5 | Cylindrical grip | Chips can | 1 | 1 | 1 | 1 | | 1 | 1 |
|  | 6 | Diagonal volar grip | Phillips screwdriver | 0.5 | 0.5 | 0.5 | 1 | | 1 | 1 |
|  | 7 | Lateral pinch | Bowl | 0.5 | 0.5 | 0.5 | 1 | | 1 | 1 |
|  | 8 | Pulp pinch | Small marker | 1 | 0.5 | 1 | 0 | | 1 | 1 |
|  | 9 | Index pointing/pressing | Timer | 1 | 1 | 1 | 1 | | 1 | 1 |
|  | 10 | Hook | Pitcher base | 0.5 | 0.5 | 0.5 | 0.5 | | 0.5 | 0.5 |
|  | 11 | Spherical grip | Softball | 1 | 0.5 | 1 | 1 | | 1 | 1 |
|  | 12 | Tripod pinch | Tuna can | 1 | 1 | 1 | 0.5 | | 0.5 | 0.5 |
|  | 13 | Extension grip | Cracker box | 0.5 | 0.5 | 0.5 | 0 | | 0 | 0 |
|  | 14 | Cylindrical grip | Coffee can | 0.5 | 0.5 | 0.5 | 0 | | 0 | 0 |
|  | 15 | Diagonal volar grip | Spatula | 0.5 | 0.5 | 0.5 | 0.5 | | 0.5 | 0.5 |
|  | 16 | Lateral pinch | XS Clamp | 1 | 1 | 1 | 1 | | 0.5 | 1 |
|  | 17 | Pulp pinch | Plastic pear | 1 | 1 | 1 | 0 | | 0.5 | 0 |
|  | 18 | Platform | Plate | 1 | 1 | 1 | - | | - | - |
|  | 19 | Hook | Wood blocks with rope | 0.5 | 0.5 | 0.5 | 0.5 | | 0.5 | 0.5 |
|  | 20 | Spherical grip | Mini soccer ball | 0.5 | 0.5 | 0.5 | 0 | | 0 | 0 |
|  | 21 | Tripod pinch | Golf ball | 1 | 1 | 1 | 1 | | 0.5 | 1 |
|  | 22 | Extension grip | Pudding box | 1 | 1 | 1 | 0 | | 0 | 0 |
|  | 23 | Cylindrical grip | Power drill | 1 | 1 | 1 | 1 | | 1 | 1 |
|  | 24 | Diagonal volar grip | Skillet | 0 | 0 | 0 | 0 | | 0 | 0 |
|  | 25 | Lateral pinch | Key | 0.5 | 0.5 | 0.5 | 0.5 | | 0.5 | 0.5 |
|  | 26 | Pulp pinch | Washer 10mm | 1 | 1 | 1 | 1 | | 1 | 0.5 |
| 2 | 1 | Hook | Skillet lid | 0.5 | 0.5 | 0.5 | 0.5 | | 0 | 0 |
|  | 2 | Spherical grip | Plastic apple | 0.5 | 0.5 | 0.5 | 1 | | 1 | 1 |
|  | 3 | Tripod pinch | Large marker | 0.5 | 1 | 0.5 | 0.5 | | 0.5 | 0.5 |
|  | 4 | Extension grip | Plate | 1 | 1 | 1 | 0 | | 0 | 0 |
|  | 5 | Cylindrical grip | Chips can | 1 | 1 | 1 | 1 | | 1 | 1 |
|  | 6 | Diagonal volar grip | Phillips screwdriver | 0.5 | 0.5 | 0.5 | 1 | | 1 | 1 |
|  | 7 | Lateral pinch | Bowl | 0.5 | 0.5 | 0.5 | 1 | | 1 | 1 |
|  | 8 | Pulp pinch | Small marker | 1 | 1 | 1 | 0.5 | | 1 | 0 |
|  | 9 | Index pointing/pressing | Timer | 1 | 1 | 1 | 1 | | 1 | 1 |
|  | 10 | Hook | Pitcher base | 0.5 | 0.5 | 0.5 | 0.5 | | 0.5 | 0.5 |
|  | 11 | Spherical grip | Softball | 1 | 1 | 1 | 0.5 | | 0.5 | 0.5 |
|  | 12 | Tripod pinch | Tuna can | 1 | 1 | 1 | 0 | | 0 | 0.5 |
|  | 13 | Extension grip | Cracker box | 0.5 | 0.5 | 0.5 | 0 | | 0 | 0 |
|  | 14 | Cylindrical grip | Coffee can | 0.5 | 0.5 | 0.5 | 0 | | 0 | 0 |
|  | 15 | Diagonal volar grip | Spatula | 0.5 | 0.5 | 0.5 | 0.5 | | 0.5 | 0.5 |
|  | 16 | Lateral pinch | XS Clamp | 1 | 1 | 1 | 1 | | 1 | 1 |
|  | 17 | Pulp pinch | Plastic pear | 1 | 1 | 1 | 0 | | 1 | 1 |
|  | 18 | Platform | Plate | 1 | 1 | 1 | - | | - | - |
|  | 19 | Hook | Wood blocks with rope | 0.5 | 0.5 | 0.5 | 0.5 | | 0.5 | 0.5 |
|  | 20 | Spherical grip | Mini soccer ball | 0.5 | 0.5 | 0.5 | 0 | | 0 | 0 |
|  | 21 | Tripod pinch | Golf ball | 1 | 1 | 0.5 | 1 | | 1 | 1 |
|  | 22 | Extension grip | Pudding box | 1 | 1 | 1 | 0 | | 0 | 0 |
|  | 23 | Cylindrical grip | Power drill | 1 | 1 | 1 | 0.5 | | 1 | 1 |
|  | 24 | Diagonal volar grip | Skillet | 0 | 0 | 0 | 0 | | 0 | 0 |
|  | 25 | Lateral pinch | Key | 0.5 | 0.5 | 0.5 | 0.5 | | 0.5 | 0.5 |
|  | 26 | Pulp pinch | Washer 10mm | 1 | 1 | 1 | 1 | | 1 | 1 |
| 3 | 1 | Hook | Skillet lid | 0.5 | 0.5 | 0.5 | 0 | | 0 | 0.5 |
|  | 2 | Spherical grip | Plastic apple | 1 | 0.5 | 1 | 1 | | 0 | 1 |
|  | 3 | Tripod pinch | Large marker | 0.5 | 0.5 | 0.5 | 0.5 | | 0.5 | 0.5 |
|  | 4 | Extension grip | Plate | 1 | 1 | 1 | 0 | | 0 | 0 |
|  | 5 | Cylindrical grip | Chips can | 1 | 1 | 1 | 1 | | 0 | 0 |
|  | 6 | Diagonal volar grip | Phillips screwdriver | 0.5 | 0.5 | 0.5 | 0.5 | | 1 | 1 |
|  | 7 | Lateral pinch | Bowl | 0.5 | 0.5 | 0.5 | 1 | | 1 | 1 |
|  | 8 | Pulp pinch | Small marker | 1 | 1 | 1 | 1 | | 0.5 | 1 |
|  | 9 | Index pointing/pressing | Timer | 1 | 1 | 1 | 1 | | 1 | 1 |
|  | 10 | Hook | Pitcher base | 0.5 | 0.5 | 0.5 | 0.5 | | 0.5 | 0.5 |
|  | 11 | Spherical grip | Softball | 1 | 1 | 1 | 0.5 | | 0.5 | 0.5 |
|  | 12 | Tripod pinch | Tuna can | 1 | 1 | 1 | 0 | | 0.5 | 0.5 |
|  | 13 | Extension grip | Cracker box | 0.5 | 0.5 | 0.5 | 0 | | 0 | 0 |
|  | 14 | Cylindrical grip | Coffee can | 0.5 | 0.5 | 0.5 | 0 | | 0 | 0 |
|  | 15 | Diagonal volar grip | Spatula | 0.5 | 0.5 | 0.5 | 0.5 | | 0.5 | 0.5 |
|  | 16 | Lateral pinch | XS Clamp | 1 | 1 | 1 | 1 | | 1 | 1 |
|  | 17 | Pulp pinch | Plastic pear | 1 | 1 | 1 | 1 | | 1 | 1 |
|  | 18 | Platform | Plate | 1 | 1 | 1 | - | | - | - |
|  | 19 | Hook | Wood blocks with rope | 0.5 | 0.5 | 0.5 | 0.5 | | 0.5 | 0.5 |
|  | 20 | Spherical grip | Mini soccer ball | 0.5 | 0.5 | 0.5 | 0 | | 0 | 0 |
|  | 21 | Tripod pinch | Golf ball | 1 | 1 | 1 | 1 | | 0.5 | 1 |
|  | 22 | Extension grip | Pudding box | 1 | 1 | 1 | 0 | | 0 | 0 |
|  | 23 | Cylindrical grip | Power drill | 1 | 1 | 1 | 1 | | 1 | 1 |
|  | 24 | Diagonal volar grip | Skillet | 0 | 0 | 0 | 0 | | 0 | 0 |
|  | 25 | Lateral pinch | Key | 0.5 | 0.5 | 0.5 | 0.5 | | 0.5 | 1 |
|  | 26 | Pulp pinch | Washer 10mm | 1 | 1 | 1 | 1 | | 0 | 1 |
