# Supplementary material for: A multiarticulate pediatric prosthetic hand for clinical and research applications
Source: Front Robot AI. 2022 Oct 28;9:1000159. doi: 10.3389/frobt.2022.1000159 (PMC9651148; doi:10.3389/frobt.2022.1000159)
Supplement: Supplementary file 2 [file Image1.PDF]

Supplementary Material

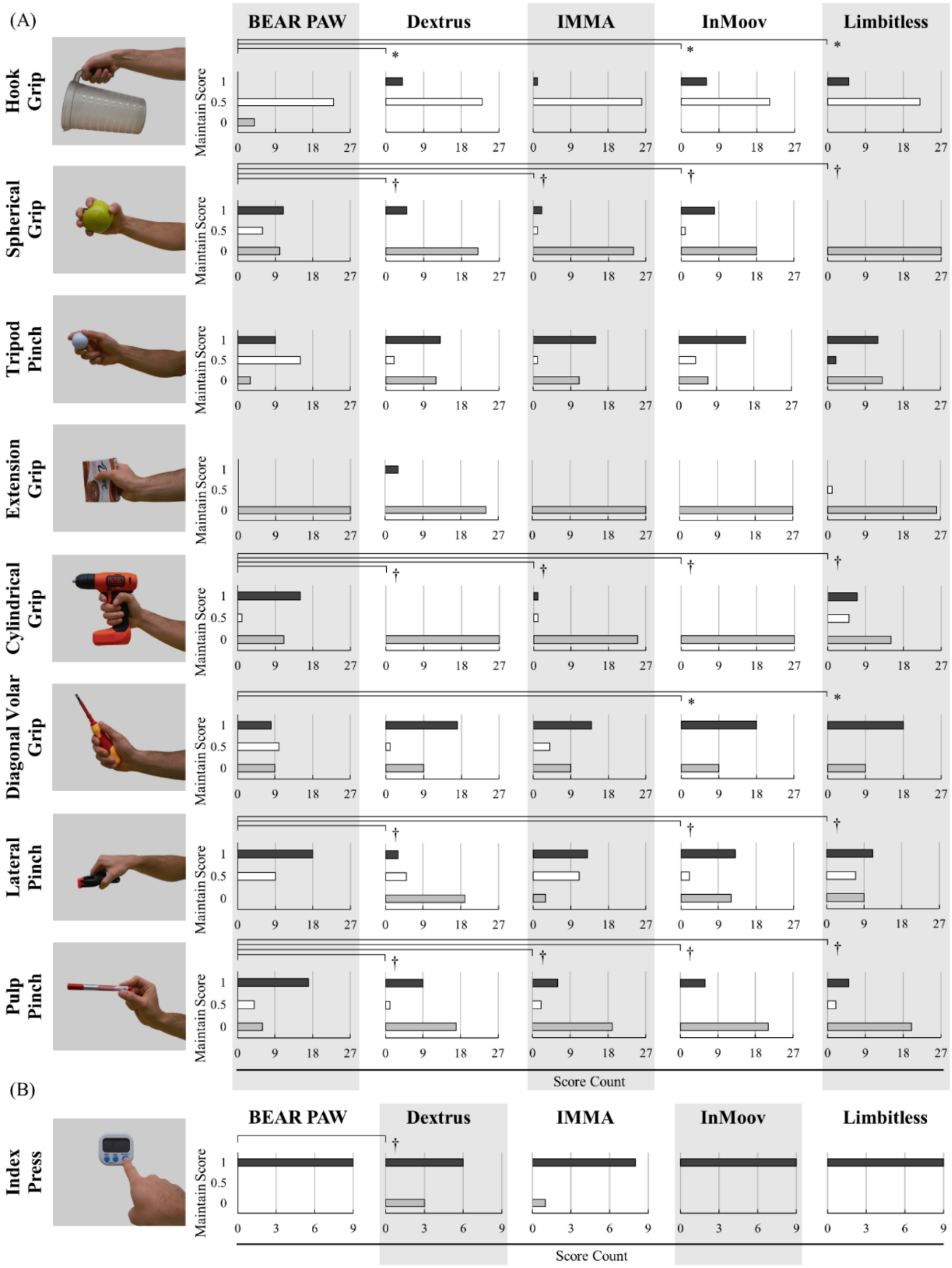

Supplementary Figure 1. BEAR PAW maintaining comparison scores for the (A) 8 grasp types and (B) 1 posture across the 4 adult hands. For each grasp type/posture, the number of times each hand scored a 1, 0.5, or 0 was plotted. *Represents when the BEAR PAW performed statistically worse. †Represents when the BEAR PAW performed statistically better.
